# Signals and Mechanisms Regulating Monocyte and Macrophage Activation in the Pathogenesis of Juvenile Idiopathic Arthritis

**DOI:** 10.3390/ijms22157960

**Published:** 2021-07-26

**Authors:** Chao-Yi Wu, Huang-Yu Yang, Jing-Long Huang, Jenn-Haung Lai

**Affiliations:** 1 Division of Allergy, Asthma, and Rheumatology, Department of Pediatrics, Chang Gung Memorial Hospital, Taoyuan 333, Taiwan; joywucgu@hotmail.com (C.-Y.W.); hjlong0182@gmail.com (J.-L.H.); 2 College of Medicine, Chang Gung University, Taoyuan 333, Taiwan; hyyang01@gmail.com; 3 Department of Nephrology, Chang Gung Memorial Hospital, Taoyuan 333, Taiwan; 4 Department of Pediatrics, New Taipei Municipal TuCheng Hospital, New Taipei City 236, Taiwan; 5 Division of Allergy, Immunology, and Rheumatology, Department of Internal Medicine, Chang Gung Memorial Hospital, Chang Gung University, Taoyuan 333, Taiwan; 6 National Defense Medical Center, Graduate Institute of Medical Science, Taipei 114, Taiwan

**Keywords:** juvenile idiopathic arthritis, macrophage, monocyte

## Abstract

Monocytes (Mos) and macrophages (Mφs) are key players in the innate immune system and are critical in coordinating the initiation, expansion, and regression of many autoimmune diseases. In addition, they display immunoregulatory effects that impact inflammation and are essential in tissue repair and regeneration. Juvenile idiopathic arthritis (JIA) is an umbrella term describing inflammatory joint diseases in children. Accumulated evidence suggests a link between Mo and Mφ activation and JIA pathogenesis. Accordingly, topics regarding the signals and mechanisms regulating Mo and Mφ activation leading to pathologies in patients with JIA are of great interest. In this review, we critically summarize recent advances in the understanding of how Mo and Mφ activation is involved in JIA pathogenesis and focus on the signaling pathways and mechanisms participating in the related cell activation processes.

## 1. Introduction: Overview of the Pathogenesis of Juvenile Idiopathic Arthritis

Juvenile idiopathic arthritis (JIA) is the leading chronic rheumatic disease of childhood and occurs in children before their sixteenth birthday with symptoms of articular inflammation for at least 6 weeks [1]. Based on the number of joints involved within the first 6 months of disease onset and extra-articular manifestations, JIA is categorized into seven subtypes according to the International League of Associations for Rheumatology (ILAR) classification [2]. Although different subtypes are heterogeneous in presentation, patients with JIA share a common phenotype of inflamed synovial membranes, which can result in growth arrest, articular deformity, and disability [3].

The etiology and pathogenesis of JIA are still elusive. Joint infiltrating inflammatory cells, residential synovial fibroblasts and osteoclasts, antibodies targeting autoantigens, and various inflammatory mediators are some of the critical players that promote the chronic inflammatory process [3,4,5,6]. In recent years, evidence suggests a distinct pathogenesis of systemic onset juvenile idiopathic arthritis (sJIA) compared to that of the oligoarticular and polyarticular forms of JIA [4]. sJIA is an autoinflammatory disease marked by universal inflammation due to a dysregulated innate immune system [2,7,8]. The abnormal activation of various phagocytes, including monocytes (Mos), macrophages (Mφs), and neutrophils, leads to a massive release of the proinflammatory mediators interleukin (IL)-1, IL-6, IL-18, and S100 proteins in sJIA [4]. On the other hand, genetic variants in human leukocyte antigen genes, the identification of cartilage-derived autoantigens, and the imbalance between regulatory T cells and autoreactive type 1 helper T (Th1)/type 17 helper T (Th17) cells in patients with oligoarticular and polyarticular JIA suggest that the disorders in this group are antigen-driven autoimmune diseases mediated by adaptive immune system disorganization [4]. However, despite the presence of autoantibodies and autoreactive lymphocytes, without the participation of innate immune mediators, the adaptive immune system cannot fully account for the development of many autoimmune diseases [7,8,9]. Indeed, an activated Mφ gene expression signature was detected in cells in synovial fluid (SF) from early-onset oligoarticular JIA patients at risk of extending arthritis joint counts [10]. Moreover, a Mo signature was found in the peripheral blood of patients with older-onset oligoarticular JIA [11] and rheumatoid factor (RF)-positive polyarticular JIA [12].

Mos/Mφs are important players in the innate immune system and are critical in coordinating the initiation, expansion, and resolution of many autoimmune diseases [8]. They possess a wide range of inflammatory, immunomodulatory, and tissue-repairing capacities via their secretion of numerous proinflammatory cytokines, growth factors, and proteolytic enzymes to stimulate and recruit effector cells to inflamed tissues [7,8,9]. A growing number of studies have highlighted the role of Mo/Mφ activation and polarization in JIA pathogenesis. In this review, we critically summarize recent advances in the understanding of how Mo and Mφ activation is involved in JIA pathogenesis and focus on the signaling pathways and mechanisms participating in the related cell activation processes.

## 2. The Origin of Synovial Resident Macrophages

The origin and ontogeny of synovial Mφs are incompletely characterized. Studies suggest that tissue-resident Mφs may be derived from the yolk sac, fetal liver Mos, or bone marrow [13,14]. While evidence has documented that tissue-resident Mφs derived from embryos can sustain themselves for a long period via local proliferation independent of hematopoietic stem cells [13,14,15], the classical mononuclear phagocytic system suggests that tissue Mφs are the final cells of the mononuclear phagocyte lineage derived from circulating Mos originating from the bone marrow [16]. Under inflammatory conditions or physiological stress, circulating Mos are believed to migrate from the bloodstream to tissues in need and to differentiate into dendritic cells or tissue-resident Mφs [16,17,18]. Potent chemokines, such as monocyte chemoattractant protein-1 (MCP-1)/C-C motif chemokine ligand (CCL)2, regulated upon activation, normal T-cell-expressed and, presumably, secreted (RANTES)/CCL5, and C-X-C motif chemokine ligand (CXCL)9 and CXCL10, were found to be significantly elevated in the SF of patients with JIA, driving the chemotactic activity of mononuclear leukocytes [19,20]. Colony-stimulating factors, such as granulocyte-macrophage colony-stimulating factor (GM-CSF) is also important in this differentiation process [21,22].

## 3. Characteristics of Monocytes and Macrophages in Juvenile Idiopathic Arthritis

### 3.1. Monocyte Subsets

Monocytes are a heterogeneous population of leukocytes circulating in the blood until they are recruited to tissues upon signaling. The expression levels of the surface molecules CD14 and CD16 determine the distinct subpopulations of human Mos [23,24]. Classical Mos are characterized by CD14^++^CD16^−^ expression and represent 80–90% of the Mos in the bloodstream of healthy individuals [25]. Nonclassical CD14^+^CD16^++^ Mos and intermediate CD14^++^CD16^+^ Mos account for a much smaller proportion of circulating Mos and can expand considerably under inflammatory conditions [25]. For example, while no significant increase in classical and intermediate Mos has been observed in patients with polyarticular JIA, the frequency of intermediate CD14^++^CD16^+^ Mos among both the circulating and synovial Mos is expanded in patients diagnosed with enthesitis-related arthritis (ERA) [26,27]. Recently, *Schmidt, Cren* and *Gaur* investigated the distribution of Mo subsets in paired SF and blood samples from patients with oligoarticular JIA and ERA. These authors discovered that while classical CD14^++^CD16^−^ Mos dominate in the circulation, intermediate CD14^++^CD16^+^ Mos were highly enriched in oligoarticular JIA and ERA patient SF [28,29,30]. As CD14^++^CD16^+^ synovial Mos can be induced by cytokine-rich SF and are found with similar patterns across Mo subsets, reports have suggested that the increased CD16 expression in these cells may likely result from the cytokine milieu of the synovial space and not the recruitment of intermediate CD14^++^CD16^+^ Mos from circulation due to their unique features [28,31]. Specifically, cytokines, such as IL-10 and transforming growth factor beta (TGFβ), in SF are potent inducers of CD16, an activating Fcγ receptor (FcγR), expression in Mos [28,29].

As demonstrated by *Macaubas* et al., the Mo lineage is expanded in patients with active sJIA [30,32]. While increased levels of CD14 and CD16 were found on sJIA Mos in both the flare and quiescence status, the distribution of the CD14^+^CD16^+^ Mo subsets was not altered compared to that in healthy controls [30,32]. Both classical CD14^++^CD16^-^ Mos and CD14^+^CD16^+^ Mos are activated in sJIA [30]. The increased expression of CD14, a pattern recognition receptor that binds lipopolysaccharide (LPS) and other microbial molecules, on sJIA Mos was proposed by *Srivastava* et al. to contribute to the apoptosis resistance of Mos [33].

Seemingly important players in JIA, intermediate Mos are noted for their high surface levels of class II molecules, CD40, CD54, and CD74, which are capable of inducing T cell stimulation and proliferation [34,35]. Upon encountering damage-associated molecular patterns or pathogen-associated molecular patterns, such as LPS, these Mos preferentially produce IL-1β, IL-6, and tumor necrosis factor (TNF)α [36,37]. Furthermore, in response to vascular endothelial growth factor (VEGF), CD14^+^CD16^+^ Mos form cell clusters and exhibit proangiogenic behavior [38]. In a rheumatoid arthritis (RA) study, a coculture of intermediate CD14^++^CD16^+^ Mos from arthritis patients with naïve T cells skewed the T cells toward pathogenic Th17 cells via the production of IL-23. The increased frequency of IL-17-producing natural killer (NK) cells, CD4, and gamma-delta T cells in patients with ERA was also recently proposed to result from the expansion of intermediate CD14^++^CD16^+^ Mos due to their role as the major producer of IL-23, a key cytokine in the pathogenesis of ERA [26].

### 3.2. Monocyte/Macrophage Polarization

Plasticity and heterogenicity are the hallmarks of Mos/Mφs. Polarization is believed to influence disease progression by altering effector function [37] and has been linked to osteoclastogenesis in RA, to disease severity in osteoarthritis, and to distinct JIA subtypes [29,34,39,40,41]. Demonstrated by the acquisition of distinct functional characteristics directed by the immunological microenvironment and tissue milieu, polarized Mos/Mφs are referred to as classically activated (M1) or alternatively activated (M2) Mφs, mirroring the Th1/Th2 nomenclature [42]. Based on the induction of cytokines and clinical features involving tissue repair, angiogenesis, and immune regulation, alternatively activated Mφs have been further subcategorized [39,43] (Table 1).

The polarization pattern of Mos/Mφs and its consequences in patients with oligoarticular JIA remain largely unclear. In a recently published report, *Schmidt* et al. suggested that the inflammatory features of those who suffered from oligoarticular JIA may not fit into the traditional dichotomous polarization categories and should be considered according to their unique pattern [31]. Specifically, compared to circulating Mos, the synovial Mos were polarized with a mixed classically and alternatively activated pattern. This was evidenced by the increased expression of the surface molecules CD40, CD86, and CD206 and by mRNA profiling showing upregulated CD80, signal transducer and activator of transcription (STAT)1, CXCL10, CD206, CCL18, and peroxisome proliferator-activated receptor gamma (PPARγ) expression, but not CD163 or STAT6 expression [31]. Correspondingly, a similar heterogeneous mixture of polarized Mφs is also seen in RA synovium and SF [40,41,44]. Interestingly, even though IL-1β, IL-6, IL-8, and IL-10 were found in JIA SF, SF alone did not induce the in vivo polarization pattern observed [31]. It is therefore hypothesized that Mos might obtain their polarization pattern, at least partially, from an extra-articular environment before migrating to the synovial space [31]. Similarly, the polarization of Mos in sJIA is highly dependent on environmental stimuli [45,46]. Although the molecular mechanism remains to be elucidated, a mixed polarization phenotype was noted in Mos isolated from patients with sJIA, possibly through interferon (IFN)/STAT signaling and the activity of small noncoding RNAs, or microRNAs [30,45,47,48].

Immunoregulatory Mφ activity was found in ERA patients, evidenced by the expression of CD163 [26,47]. CD163 is a scavenger receptor that is enhanced in response to IL-10 [48] and has been linked to the pathogenesis of autoimmune disorders in adults, especially spondyloarthropathies [49]. An increased production of the proinflammatory cytokines IL-1β, IL-6, and TNFα and the immunoregulatory mediators IL-10 and nitrogen oxide was elevated upon the cross-linking of CD163 [37]. In addition to the proinflammation tendency, an immunoregulatory effect of CD163^+^ Mφs has been suggested as soluble CD163 can suppress the activation and proliferation of T lymphocytes upon stimulation [50]. The anti-inflammatory heme metabolite production results from CD163 hemoglobulin transportation and the production of IL-10 together, also attributed to the immunosuppressive Mφ phenotype [51,52]. Collectively, Mφs with high CD163 expression have been hypothesized to downregulate the inflammatory response in the late inflammation phases [47].

## 4. Mediators Directing Monocyte/Macrophage Activation and Polarization

### 4.1. Cytokines

Cytokines play a critical role in the activation and polarization of Mos/Mφs. As summarized in Table 1, while GM-CSF, TNFα, and IFNγ drive these cells to a proinflammatory state, IL-10 is especially important for the immunoregulatory Mo/Mφ polarization in JIA.

#### 4.1.1. GM-CSF

GM-CSF, a hemopoietic growth factor, is secreted by myeloid cells, T and B lymphocytes, and tissue residential cells, such as fibroblasts, chondrocytes, osteoblasts, and epithelial and endothelial cells. GM-CSF interacts with the GM-CSF receptor and activates the Janus kinase (JAK)2-STAT5- suppressor of cytokine signaling (SOCS) as well as mitogen-activated protein kinases (MAPKs), phosphatidylinositol 3 kinase, and nuclear factor kappa-light-chain-enhancer of activated B cells (NFκB), resulting in the activation of tissue-resident cells and the recruitment of inflammatory cells [53]. The production of IL-6 and IL-23 upon GM-CSF receptor signaling activates T cells and promotes the differentiation of Th17 cells to induce additional GM-CSF and IL-17 secretion, forming a feedback loop [53]. While the role of GM-CSF in JIA is not as well studied as that in RA [53], the frequency of GM-CSF-producing T helper cells was significantly enriched among SF mononuclear cells, and the culturing of Th17 cells in the presence of IL-12 resulted in upregulation of GM-CSF and IFNγ, recapitulating the phenotype of GM-CSF-expressing cells within the JIA joints [54].

#### 4.1.2. TNFα

TNFα is a pleiotropic cytokine that promotes the expression of adhesion molecules, inflammatory cytokines, prostaglandin E2, collagenase, and collagen by synovial cells [55]. As Mos/Mφs are the main producers of TNFα, its production serves as an autocrine stimulator and a potent paracrine inducer of inflammatory cytokines, including IL-1, IL-6, IL-8, and GM-CSF [56,57,58]. TNFα triggers NFκB and MAPK activation and/or cell death via apoptosis or necroptosis via either death domain adaptor or TNF receptor associated factor (TRAF) depending on the distinct receptor type (TNFαR1 and TNFαR2) to which it binds [59]. Significantly higher TNFα expression was found in JIA patient plasma without stratifying for disease activity or JIA subtype, and increased levels of serum TNFα and soluble TNFαR1 and TNFαR2 were noted in patients with active sJIA, including those complicated with macrophage activation syndrome (MAS) [20,60]. Upon treatment with TNF blocking agents, the inflammatory Mφs were shifted toward an immunoregulatory phenotype with reduced production of inflammatory cytokines and increased phagocytosis and apoptosis [61,62,63,64].

#### 4.1.3. IFNγ

IFNγ, the sole member of type II IFNs, is produced by multiple cell types, including T cells (Th1 cells in particular), B cells, Mos/Mφs, and NK cells [65]. It regulates more than 9000 genes to orchestrate the production of cytokines and reactive oxygen species, enhance antigen presentation, cellular differentiation, and Mφ activation as well as cell growth and survival [65]. Upon binding to its receptor, JAK1, JAK2, and STAT1 are recruited and activated via phosphorylation to modulate gene transcription [65]. An enhanced responsiveness to IFNγ stimulation was reported with increased STAT1 and/or STAT3 phosphorylation in classical Mos in patients with polyarticular JIA [27]. Although no obvious differences in IFNγ levels were noted in oligoarticular, polyarticular, and sJIA patients under distinct activity statuses when compared to controls [20], altered RNA profiling with upregulation of the IFNγ pathway and increased expression of tripartite motif containing eight was identified in a distinct subpopulation of bone marrow Mφs in sJIA patients complicated with MAS [66]. Interestingly, *Macaubas* et al. recently discovered impaired IFN/STAT1 signaling in sJIA Mos during active disease, skewing them away from a classically activated phenotype [67]. However, this hyporesponsiveness to IFN was restored and inverted in treated, quiescent subjects [67].

#### 4.1.4. IL-10

IL-10 is a critical cytokine in the negative feedback loop that limits inflammation and increases phagocytosis [68]. IL-10 induction often occurs along with proinflammatory cytokines, although pathways that induce IL-10 often negatively regulate inflammatory effects [68]. The rapid transcription of IL-10 mRNA was reported during proinflammatory Mo/Mφ activation [61]. IL-10 inhibition resulted in a dramatic deprivation in immunoregulatory surface markers with a concomitant increase in inflammatory surface markers [61]. Upon the binding of IL-10 and its receptors (IL10RA and IL10RB), STAT3 signaling is induced through phosphorylation by JAK1 and tyrosine kinase 2 [61,68].

### 4.2. Toll-Like Receptor Signaling

Toll-like receptors (TLRs) belong to the group of protein recognition receptors and are type I transmembrane proteins serving as the first-line defense against microbes. They recognize both invading pathogens and endogenous danger molecules and are crucial in bridging innate and adaptive immunity [69]. Upon ligand interaction, the dimerization of most TLRs triggers the recruitment of myeloid differentiation primary response protein 88 (MyD88), which interacts with IL-1R-activating kinase (IRAK)4 and IRAK1/IRAK2, activates TRAF6, and enhances the transcriptional activity and production of a number of proinflammatory cytokines and chemokines through the NFκB protein complex [69]. In addition, TLR3 and TLR4 interact with TIR-domain-containing adapter-inducing interferon- (TRIF)β, activate TRAF3, and promote type 1 IFN production [69].

Studies in experimental models have documented the ability of microbial TLR ligands to trigger arthritis in animals [70]. Increased TLR2 and TLR4 expression was found on ERA peripheral blood mononuclear cells (PBMCs) and SF Mos [71], and an increased TLR/IL-1R signature and TLR2 expression were revealed by analyzing gene expression in PBMCs from patients with sJIA [72,73]. Endogenous TLR ligands, such as S100A8/A9 (calprotectin), S100A12, high mobility group box 1, and serum amyloid A, are significantly elevated in JIA cases, particularly in sJIA [74,75,76], and are likely to lead to disease progression [77]. Specifically, the binding of TLR4 with S100A8/A9 on Mos/Mφs was found to induce the transcription of chemokine IFNγ inducible protein 10 (IP-10)/CXCL10 via TRIF signaling [78]. Local injection of exogenic S100A8 into the knee joints of mice resulted in enhanced expression of the FcγR on synovial Mφs via TLR4 [79]. Moreover, in experimental models, S100A8/A9 has been shown to play an essential role in the induction of autoreactive CD8+ T cells and the development of systemic autoimmunity [80]. Interestingly, TLR signaling can be altered according to different disease statuses. During disease remission and off treatment, dysregulated responses to TLR4, TLR8, and TLR7 stimulation were observed in sJIA Mos [81].

### 4.3. Autoantibodies and Immunocomplexes

By definition, the presence of autoantibodies, RF and anti-citrullinated protein antibodies (ACPA) is the hallmark of RF-positive JIA [82]. RFs are autoantibodies targeting the fragment crystallizable portion of immunoglobulins, mostly in the IgM isotype [83]. ACPAs interact with a variety of citrullinated proteins and are associated with greater articular damage and a poorer response to therapy [83]. They interact with self-antigens, form immunocomplexes (ICs) and induce the production of TNFα or other cytokines by PBMCs via FcγR engagement [5]. Cellular responses were synergized and augmented when RF and ACPA were both presented [5]. In addition to FcγR engagement, ACPA ICs also stimulate Mφs via the dual engagement of TLR4/MyD88 and FcγR for the production of TNFα [84]. Moreover, ACPAs can directly interact with surface-expressed citrullinated proteins on RA Mos to facilitate inflammatory responses through the c-Jun N-terminal kinase and NFκB pathways [84].

### 4.4. Hypoxia

The hypoxic nature of JIA synovium and the induction of chemokine CCL20 in JIA synovial Mos within the hypoxic milieu of inflamed joints suggests that hypoxia likely increases inflammatory cell infiltration and contributes to the development of local inflammation [85,86]. As the expression of hypoxia-inducible factor (HIF)-1α and HIF-2α was constitutively detected in Mos recruited to inflamed joints in JIA patients, the ability of these key regulators to alter metabolic reactions and protein transcription potentially impacts the activation and polarization of Mos/Mφs in JIA [85]. Specifically, HIF-1α increases the transcription of glycolytic enzymes and promotes the production of the proinflammatory cytokine IL-1β [87]. In addition, decreased myeloid cell joint infiltration and delayed disease progressions were observed in an inflammatory arthritis model utilizing HIF-1α-deficient Mφs [88]. Interestingly, Raggi et al. discovered that synovial Mφs in the hypoxic inflamed joints of oligoarticular JIA patients express high surface levels of triggering receptors expressed on myeloid cells (TREM)-1 [89]. As a hypoxia-inducible gene, TREM-1 reverses the immunoregulatory-polarizing effect of hypoxia and drives proinflammatory reprogramming in a hypoxic microenvironment [89]. Hypoxic synovial Mos in JIA also release VEGF and osteopontin. These proangiogenic mediators within inflamed joints drive neoformation of blood vessels through stimulation of epithelial cell survival, proliferation, and chemotaxis, as well as monocytic cell recruitment and activation [85].

### 4.5. MicroRNAs

The polarization of Mos/Mφs in sJIA was also found to be driven by epigenetic factors, such as negative transcriptional regulation by microRNAs [50,52,90]. Through a microRNA array analysis comparing the expression of microRNAs in Mos from patients with inactive sJIA, active sJIA, new-onset sJIA, and active polyarticular JIA, *Schulert* et al. discovered that miR-125a-5p was highly upregulated in active and new-onset sJIA patients and correlated with their systemic features but not the degree of joint involvement [45]. Through microRNA overexpression and inhibition assays, miR-125a-5p was shown to drive Mos toward alternatively activated polarization with an enhanced M2b phenotype, in line with the Mos observed in sJIA in clinical settings [45]. Further investigation suggested that miR-125a-5p and miR-181c overexpression in patients with active sJIA significantly reduced the expression of CD163 on Mφs [91]. Specifically, miR-181 targets CD163 mRNA for degradation and miR-125a-5p decreases IL10RA, the receptor required for IL-10-mediated CD163 expression [91]. Moreover, miR-155 promotes Mφ proinflammatory polarization and suppresses alternatively activated features [92]. miR-155 targets SOCS1 transcription, altering cytokine and surface molecule expression [92]. Compared to controls or patients with clinically inactive sJIA, miR-155 is increased in Mos from children with active sJIA [45]. Together, these data explain how microRNAs aid sJIA Mos toward polarization to an immunoregulatory phenotype [91]. In addition, significantly higher levels of plasma miR-233, a microRNA regulating inflammation, cell differentiation, and oncogenesis, were found in JIA patients. miR-233 promotes the polarization of Mφs toward an immunoregulatory phenotype via direct targeting of PBX/Knotted 1 Homeobox 1 and controls Mφ inflammatory responses by inhibiting NOD-, LRR-, and pyrin domain-containing protein 3 (NLPR3) inflammasome activity [93,94].

## 5. Effect of Monocyte/Macrophage-Produced Cytokines and Chemokines

Activated Mos and Mφs in JIA synovial space secrete a variety of proinflammatory cytokines, including TNF-α, IL-1β, IL-6, IL-12, IL-18, and IL-23 [12,22,95]. While the levels of these mediators may vary between different JIA subtypes [96], the interactions of these cytokines and chemokines with leukocytes and residential mesenchymal cells, such as synovial fibroblasts, chondrocytes, and osteoclasts contribute JIA pathogenesis [90].

### 5.1. TNFα

The importance of TNFα in inflammatory arthritis has been highlighted in numerous clinical observations and experimental settings. In addition to its autocrine feedback role in propagating an inflammatory response [56,57,58], TNFα is capable of stimulating fibroblasts to express adhesion molecules, such as intracellular adhesion molecule 1 (ICAM-1), to enhance leukocyte adhesion [56]. Considering that bone destruction is also a hallmark of JIA, TNFα stimulates osteoclast differentiation via NFкB signaling and upregulates several proinflammatory cytokines, including receptor activator of NFκB (RANK), leading to increased RANK/RANK ligand (RANKL) signaling and osteoclast activity [97,98]. Moreover, aside from its soluble form, as a transmembrane protein, TNFα on both Mφs and fibroblasts has been shown to induce arthritis in transgenic mice [95]. Currently, the success of treating JIA patients with various biologics interfering with TNFα signaling has confirmed the importance of TNFα in the pathogenesis of JIA [99,100,101].

### 5.2. IL-1β

Innate proinflammatory cytokines, such as IL-1, IL-6, IL-18, and TNF, account for many of the features observed in sJIA [46]. Specifically, IL-1β, a pleiotropic proinflammatory cytokine, can upregulate its own transcription as well as that of IL-6 [102]. Moreover, a possible positive feedback loop involving IL-1β and S100 proteins has been proposed to contribute to the perpetuation of chronic inflammation in sJIA [103]. Interestingly, *Cepika* et al. discovered that the stimulation of Mos in patients with sJIA resulted in an increase in activin receptor signaling, which in turn inhibited IL-1β secretion without altering the accumulation of intracellular IL-1β within sJIA Mos [81]. Moreover, IL-1β induces the expression of ICAM-1 on synovial fibroblasts and activates osteoclasts to display a high degree of resorbing activity [104,105]. The critical role of IL-1β in JIA has been demonstrated by the use of the IL-1β blocking agents anakinra and canakinumab in sJIA treatment with success [106,107,108,109].

### 5.3. IL-6

IL-6 is a multifunctional cytokine that drives JIA development via immune response regulation, hematopoiesis, and bone metabolism [98]. IL-6 elevation in active sJIA modulates the levels of proteases and their regulators, such as *matrix metalloproteinase (MMP)*-9 and its tissue inhibitors of metalloproteinases-1, in synoviocytes and chondrocytes [110,111]. IL-6 also alters the cytokine profile in JIA synoviocytes in both a proinflammatory and anti-inflammatory manner [20]. Moreover, IL-6 plays a role in T cell survival and proliferation and promotes the differentiation of Th17 cells [112]. Interestingly, two distinct groups of sJIA patients with specific clinical features were identified based on their IL-6 and IL-18 levels [113]. Patients with dominant IL-6 cytokines have more severe joint disease, and those with dominant IL-18 cytokines are more likely to develop MAS [114]. As the severity of experimental inflammatory arthritis is greatly suppressed in IL-6^-/-^ transgenic mice [115], a promising result was also noted by applying tocilizumab, an IL-6 receptor targeting agent, for the treatment of polyarticular JIA and sJIA [116,117].

### 5.4. IL-18

In patients with active sJIA, the level of IL-18 is significantly elevated [118]. As documented in the RA joint, IL-18 may induce an inflammatory process because it promotes leukocyte extravasation by upregulating endothelial cell adhesion molecules, releasing chemokines from RA synovial fibroblasts, and serving as a chemoattractant for various leukocytes [119]. Moreover, IL-18 helps develop and maintain the inflammatory pannus by binding and activating endothelial cells and inducing synovial fibroblasts to produce angiogenic chemokines and VEGF, contributing to the vascularity of inflamed pannus [119,120].

### 5.5. IL-12/IL-23

IL-12 and IL-23 are mainly produced by inflammatory myeloid cells and are critical mediators influencing the differentiation of Th1 and Th17 cells [121]. While IL-23 is specifically important for the development of Th17 and plays a pathogenic role in seronegative spondyloarthropathies, including ankylosing spondylitis (AS), psoriatic arthritis, and ERA [122,123], IL-12 initiates the differentiation of naïve CD4 T cells to Th1 cells and promotes a shift of Th17 cells toward Th17/Th1 and non-classic Th1 cells [124]. These cells are all involved in the pathogenesis of JIA [4,125,126].

Specifically, increased numbers of Th1 and Th17 were reported in the synovial space of patients with oligoarticular JIA and ERA [124,127]. IL-17A and TNFα produced by Th17 and Th1 cells promotes the release of IL-6 and IL-8 by synoviocytes and endothelial cells in the joint space [128]. These cytokines induce expression of adhesion molecules, enhance leukocytes recruitment, and maintain joint inflammation [129]. Moreover, IL-17 produced by Th17 cells stimulates the release of MMPs by synovial fibroblasts and increases osteoclast differentiation, leading to cartilage breakdown and bony erosion [128]. Notably, IL-17 is capable of maintaining articular inflammation independent of TNFα once the arthritis is initiated [125].

### 5.6. IL-10

IL-10 is a potent cytokine that represses proinflammatory responses and limits unnecessary tissue damage caused by inflammation [126]. IL-10 hinders Mo trafficking into synovial tissue through the downregulation of ICAM-1 expression on synovial cells; suppresses the release and function of the proinflammatory cytokines IL-1, TNFα, and IL-6; and reverses cartilage degradation by mononuclear cells in patients with RA [126,130]. As IL-10 polymorphism confers the susceptibility to JIA [131], animal studies have suggested that insufficient IL-10 production is a mechanism underlying the pathogenesis of sJIA [132].

### 5.7. Chemokines

Increased levels of chemokines were found in JIA SF, favoring the migration of Mos to the inflamed tissue while promoting their activation and differentiation. While synovial fibroblasts are important sources of proinflammatory cytokines and chemokines [133], synovial Mos/Mφs also secrete a variety of chemokines. CCL2/MCP-1 principally recruits Mos, dendritic cells, and memory T cells to sites of inflammation [19,134]. The expression of CCL18 is upregulated in JIA synovial Mos and is capable of recruiting cells of the adaptive immune system to maintain homeostasis [31,135]. Secreted CCL20, so-called macrophage inflammatory protein-3, is also important in driving Th17 recruitment to the inflamed joints in patients with JIA [92,136,137]. Other chemokines, such as CXCL8 and CXCL10, mediate the recruitment of neutrophils, T lymphocytes, NK cells, dendritic cells, and Mos/Mφs into the joint space, coordinating an inflammatory response [31,96].

### 5.8. Vascular Endothelial Growth Factor

VEGF is the most critical regulator of angiogenesis and mediates inflammatory and bone-destructive activities [138,139]. Synovial Mos/Mφs in the hypoxic microenvironment are a source of VEGF and the concentration of VEGF was significantly increased in the SF of JIA patients compared with that in the serum [85,140]. While a number of studies have clearly documented a reduction in disease severity and synovial angiogenesis when treating RA patients with VEGF-blocking agents [141], VEGF also serves as a useful marker for assessing the disease activity of oligo/polyarticular JIA during the remission phase [136]. The tapering of medication in oligo/polyarticular JIA is recommended if the level of VEGF remains low [136].

## 6. Available Treatments and Emerging Therapeutic Opportunities

Based on the clinical relativity and the current knowledge on the pathogenesis of Mos/Mφs in JIA, different strategies have been explored or are currently under investigation for JIA patients and/or have been tested in arthritic animal models. The induction of anti-inflammatory human Mos is a unique property of glucocorticoids [137]. In arthritic rodent models, the local injection of triamcinolone acetonide strongly enhanced Mφ activation toward an immunoregulatory phenotype [142]. This was supported by the enhanced surface expression of CD163 and enhanced IL-10 expression at the mRNA level in ex vivo Mφs [142]. Methotrexate (MTX), a commonly prescribed disease-modifying antirheumatic drug (DMARD) for JIA, exclusively modulates gene expression in proinflammatory Mφs polarized by GM-CSF [143]. Further study revealed that MTX increases the expression of A20, an NFκB suppressor, which inhibits TLR signaling in GM-CSF-polarized Mφs [144]. Moreover, MTX has been reported to dampen the production of TNFα and IL-12 in classically activated Mφs [145]. On the other hand, MTX enhances IL-10 synthesis and inhibits NFκB signaling on immunoregulatory Mφs [145]. Chronic exposure to sulfasalazine, another widely used DMARD, markedly sensitized human Mos/Mφs to steroid treatment via the NFκB signaling pathway, upregulated glucocorticoid receptor α and glucocorticoid expression levels and induced apoptosis [146].

Biological therapies targeting the Mo/Mφ-producing cytokines TNFα, IL-1β, and IL-6 are now recommended as the standard of care for JIA patients with an advanced disease course [147]. While these biologics significantly dampen the proinflammatory response mediated by Mos/Mφs, anti-TNF agents not only inhibit the inflammatory functions of Mφs but also favor the resolution of inflammation by inducing cellular polarization toward alternative features involving the IL-10/STAT3 axis [61]. A decline in Mφs was clearly noted in the inflamed joints of mice shortly after the introduction of infliximab, a TNFα blocking agent [148,149]. Moreover, tocilizumab, an anti-IL6 receptor antibody, shifts Mos/Mφs toward an anti-inflammatory phenotype and induces the apoptosis of Mos [150,151]. Tofacitinib, a small molecule JAK inhibitor, was recently shown to reduce disease flares and improve disease activity and physical function in patients with polyarticular JIA [152]. It abrogates TNF induced STAT1 activation and inhibits proinflammatory mediator production [153].

The effectiveness of existing anti-rheumatic regimens and novel therapeutic agents targeting Mos/Mφs in patients with inflammatory arthritis has been investigated. A brief summary of Mo/Mφ-related therapies is shown in Table 2.

## 7. Conclusions and Future Perspectives

**Figure 1:**
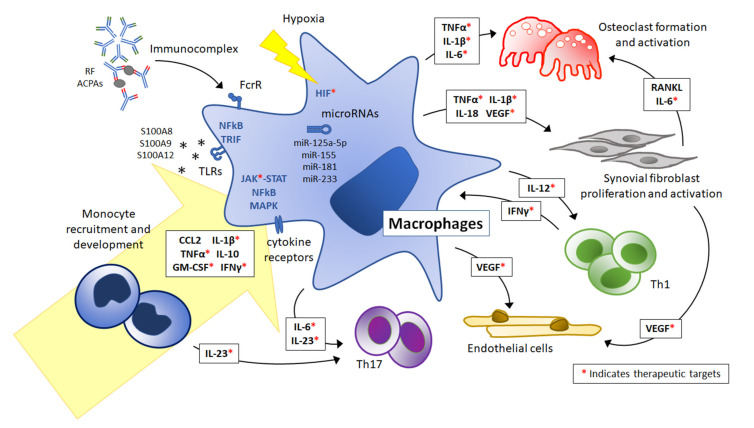
Therapeutic targets related to monocyte and macrophage activation in juvenile idiopathic arthritis. Monocytes (Mos) were recruited to the joints by CCL2 and differentiated into tissue macrophages (Mφs) under inflammatory conditions. IL-1β, TNFα, IFNγ, GM-CSF, and IL-10 are especially important for the differentiation process. Upon stimulation with cytokines, immunocomplexes, TLR ligands, the hypoxic microenvironment, and microRNAs, the mixed polarization of Mφs toward an inflammatory phenotype was mediated via the JAK-STAT, MAPK, NFκB, TRIF, and HIF signaling pathways. Proinflammatory cytokines, including IL-1β, IL-6, IL-12, IL-18, IL-23, and TNFα were secreted to stimulate activation of osteoclasts and synovial fibroblasts and to promote T lymphocyte polarization. VEGF drives synovial angiogenesis, contributing to JIA pathogenesis. Available therapeutic targets are marked with red asterisks *. Abbreviations: RF, rheumatoid factor; ACPAs, anti-citrullinated protein antibodies; CCL2, monocyte chemoattractant protein 1; TLRs, Toll-like receptors; FcγR, Fc gamma receptor; JAK, Janus kinase; STAT, signal transducer and activator of transcription; MAPKs, mitogen-activated protein kinases; TRIF, TIR-domain-containing adapter-inducing interferon; NFkB, nuclear factor kappa-light-chain-enhancer of activated B cells; HIF, hypoxia-inducible factor; Th1, type 1 T helper cells; Th17, type 17 T helper cells; RANKL, receptor activator of nuclear factor kappa beta ligand.

The present review summarizes recent understanding of the phenotype of Mos/Mφs and the mechanisms regulating their activation to elicit inflammation in patients with JIA. As discussed, the activity and phenotype of Mos/Mφs play an important role in pathogenesis across different JIA subtypes. Recruited to the joints by chemokine MCP-1/CCL2 and RANTES, Mos infiltrate the inflamed synovium and add to the tissue-resident Mφ pool orchestrating a local and global inflammatory reaction resulting in tissue damage. Upon the stimulations of cytokines, immunocomplexes, TLR ligands, the hypoxic microenvironment, and microRNAs, a mixed polarization of Mφs toward an inflammatory phenotype was mediated via the JAK-STAT, MAPK, NFκB, TRIF, and HIF signaling pathways. Proinflammatory cytokines, including IL-1β, IL-6, IL-12, IL-18, IL-23, and TNFα were secreted to stimulate activation of osteoclasts and synovial fibroblasts and to promote T lymphocyte polarization. VEGF, in addition, drives synovial angiogenesis, contributing to JIA pathogenesis (Figure 1).

While investigations into strategies targeting Mo recruitment, Mo/Mφ polarization, cell depletion, and cytokine blockade have been recently performed (Table 2), uncovering the heterogenicity and regulatory mechanisms of Mo/Mφ in JIA pathogenesis is crucially needed for the development of novel approaches aiming at Mo/Mφ for the control of the disease.

## Figures and Tables

**Table 1:** Characteristics of monocyte and macrophage polarization in humans.

Macrophages	Classically Activated (M1)	Alternatively Activated (M2)		
		M2a	M2b	M2c
Mediators of polarization	LPS, IFNγ, TNFα, GM-CSF	IL-4, IL-13	ICs + TLR/IL-1β	IL-10, TGFβ, steroid
Surface markers	CD68, CD80, CD86, IL-1R, TLR2, TLR4, iNOs, IFNγR, MHC-II^high^	CD200R, CD206/MMR, IL-1RII, Dectin-1, MHC-II^low^	CD86, MHC-II^low^	CD163, TLR1, TLR8
Transcription factors and cellular markers	NFκB, STAT1, STAT5, IRF3, IRF5	IRF4, PPARγ, STAT6	IRF4, SOCS3	IRF4, SOCS3
Produced cytokines	IL-1α, IL-1β, IL-6, IL-12, IL-18, TNFα, M-CSF	IL-10, TGFβ, IL-1Ra	IL-1β, IL-6, IL-10, TNFα	IL-10, TGFβ
Produced chemokines	CXCL9, CXCL10, CXCL11	CCL17, CCL18, CCL22, CCL24	CCL1, CCL20, CXCL1, CXCL2, CXCL3	CCL16, CCL18
Features	Proinflammatory, microbicidal, and tumoricidal	endocytic activity, tissue remodeling, and repair	immunoregulation	immunoregulation

**Table 2:** Therapeutic agents targeting monocytes/macrophages and their functional activities.

Agent	Targets	Actions	Developmental Stage	Ref.
Etanercept	TNF receptor	Blocks TNFα signaling; shifts Mos/Mφs toward an anti-inflammatory phenotype and induces apoptosis of Mos/Mφs	JIA–marketing	[61,62]
Adalimumab	TNF	Blocks TNFα signaling; shifts Mos/Mφs toward an anti-inflammatory phenotype; induces apoptosis of Mos/Mφs and reduces Mo migration into the joint	polyarticular JIA–marketing	[63,149,154]
Infliximab	TNF	Blocks TNFα signaling; shifts Mos/Mφs toward an anti-inflammatory phenotype and induces apoptosis of Mos/Mφs; increases circulating nonclassical Mos and decreases circulating classical Mos; reduces CCR2 and CXCR4 expression on the nonclassical Mo subpopulation	JIA–marketing	[62,63,64]
Certolizumab	TNF	Blocks TNFα signaling; induces HO-1 mRNA and protein production in Mos; inhibits IL-1β production at the mRNA and protein level upon LPS stimulation	polyarticular JIA–phase III	[154]
Anakinra	IL-1β receptor	Blocks IL-1β signaling	sJIA–marketing	[109]
Canakinumab	IL-1β	Blocks IL-1β signaling	sJIA–marketing	[106,107]
Rilonacept	IL-1β/IL-1α	Blocks IL-1 signaling; skews Mos toward an alternatively activated phenotype	sJIA–phase II	[155]
Tocilizumab	IL-6 receptor	Blocks IL-6 signaling; shifts Mos/Mφs toward an anti-inflammatory phenotype and induces apoptosis of Mos	sJIA/polyarticular JIA–marketing	[150,151]
Sarilumab	IL-6 receptor	Blocks IL-6 signaling	polyarticular JIA–phase II	[156]
Ustekinumab	IL-12/IL-23	Blocks IL-12/IL-23 signaling	psoriatic arthritis–marketing	[157]
Secukinumab	IL-17A	Blocks IL-17 signaling; decreases serum IL-6, S100A8, S100A9, VEGF, TNFα, osteopontin, and MMP	ERA/juvenile psoriatic arthritis –phase III	[158]
Ixekizumab	IL-17A	Blocks IL-17 signaling	ERA/juvenile psoriatic arthritis –phase III	[159]
Emapalumab	IFNγ	Blocks IFNγ signaling	sJIA–phase II	[160]
Abatacept	CTLA-4	Blocks ACPA and RF mediated cytokine production in human Mφs; modulates proinflammatory Mφ responses upon cytokine-activated T cell and TLR stimulation	Polyarticular JIA–marketing	[161,162]
Tofacitinib	JAK1/JAK3	Small molecule that abrogates TNF- induced STAT1 activation; inhibits proinflammatory mediator production	polyarticular JIA–marketing	[153]
Baricitinib	JAK1/JAK2	Decreases expression of the inflammatory IP-10 and increases IL-10 production	JIA–phase III	[163]
Upadacitinib	JAK1	Selectively targets JAK1 dependent disease drivers such as IL-6 and IFNγ	Polyarticular JIA–phase I	[164]
Mavrilimumab (CAM-3001)	GM-CSF receptor α	Blocks GM-CSF signaling and classically activated polarization	RA–phase IIb	[165]
Otilimab (MOR103)	GM-CSF	Blocks GM-CSF signaling and classically activated polarization	RA–phase III	[166]
Givinostat (ITF2357)	histone deacetylase inhibitor	Prevents LPS-induced TNFα gene transcription and secretion of IL-1β	JIA–phase II	[167]
Gamma-linolenic acid	n-6 polyunsaturated fatty acids	Inhibits inflammatory responses through inactivation of NFκB and AP-1 by suppressing oxidative stress and the ERK and JNK signal transduction pathways in LPS-induced Mφs	JIA-phase I	[168]
Sinomenine	plant alkaloid	Attenuates CD11b^+^F4/80^+^CD64^+^ resident Mφs in the synovial tissue and reduces number of CD14^+^CD16^+^ circulating Mos	Herbal medicine	[169]
Thapsigargin	inhibitor of SERCA	Decreases the number of TNF-induced classically activated Mφs and increases the number of alternatively activated Mφs	preclinical	[170]
Withaferin-A	steroidal lactone	Promotes classically activated Mφ to alternatively activated Mφ repolarization	preclinical	[171]
Berberine	antimicrobial agent	Increases the proportion of alternatively activated Mφs and decreases the proportion of classically activated Mφs, downregulates HIF-1α expression in synovial Mφs	Preclinical	[172]
Ramucirumab	VEGF	Blocks VEGF signaling	preclinical	[173]
Ranibizumab	VEGF	Blocks VEGF signaling	preclinical	[174]
2-benzoyl-phenoxy acetamide	benzophenone analog	Targets VEGF and HIF-1α	preclinical	[175]
Paclitaxel (PTX)	tubulin, chemotherapy	Targets VEGF and HIF-1α	preclinical	[176]
pLVX-shRNA-HIF-1α	shRNA targeting HIF-1α	Inhibits HIF-1α and VEGF expression, leading to decreased proinflammatory cytokine expression	Preclinical	[177]
Clodronate liposomes	release chlorophosphate	Mφ depletion	Preclinical	[178]
Human umbilical cord blood-derived mesenchymal stem cells	stem cells	Polarizes naive Mφs toward an alternatively activated phenotype	preclinical	[179]

## Abbreviations

ACPA	anti-citrullinated protein antibody
AS	ankylosing spondylitis
CCL	C-C motif chemokine ligand
CXCL	C-X-C motif chemokine ligand
DMARD	disease-modifying antirheumatic drug
ERA	enthesitis-related arthritis
FcγR	Fcγ receptor
GM-CSF	granulocyte-macrophage colony-stimulating factor
HIF	hypoxia-inducible factor
IC	immunocomplex
ICAM-1	intracellular adhesion molecule 1
IL	interleukin
ILAR	International League of Associations for Rheumatology
IFN	Interferon
IP-10	IFNγ inducible protein 10
IRAK	IL-1R-activating kinase
IRF	interferon regulatory factor
JAK	Janus kinase
JIA	juvenile idiopathic arthritis
LPS	lipopolysaccharide
MAPK	mitogen-activated protein kinase
MAS	macrophage activation syndrome
MCP-1	monocyte chemoattractant protein-1
MMP	matrix metalloproteinases
Mφ	macrophage
Mo	monocyte
MTX	methotrexate
MyD88	myeloid differentiation primary response protein 88
NFκB	nuclear factor kappa-light-chain-enhancer of activated B cells
NK	natural killer
NLRP3	NOD-, LRR-, and pyrin domain-containing protein 3
PBMCs	peripheral blood mononuclear cells
PPARγ	peroxisome proliferator-activated receptor gamma
RA	rheumatoid arthritis
RANTES	regulated upon activation, normal T cell expressed and presumably secreted
RF	rheumatoid factor
RANK	receptor activator of NFκB
RANKL	RANK ligand
SF	synovial fluid
sJIA	systemic onset JIA
SOCS	suppressor of cytokine signaling
STAT	signal transducer and activator of transcription
TGFβ	transforming growth factor beta
Th1	type 1 helper T cells
Th17	type 17 helper T cells
TLR	Toll-like receptor
TNF	tumor necrosis factor
TREM	triggering receptor expressed on myeloid cells
TRAF	TNF receptor associated factor
VEGF	vascular endothelial growth factor
